# Role of domestic ducks in the emergence of a new genotype of highly pathogenic H5N1 avian influenza A viruses in Bangladesh

**DOI:** 10.1038/emi.2017.60

**Published:** 2017-08-09

**Authors:** Subrata Barman, Atanaska Marinova-Petkova, M Kamrul Hasan, Sharmin Akhtar, Rabeh El-Shesheny, Jasmine CM Turner, John Franks, David Walker, Jon Seiler, Kimberly Friedman, Lisa Kercher, Trushar Jeevan, Daniel Darnell, Ghazi Kayali, Lisa Jones-Engel, Pamela McKenzie, Scott Krauss, Richard J Webby, Robert G Webster, Mohammed M Feeroz

**Affiliations:** 1 Department of Infectious Diseases, St Jude Children’s Research Hospital, Memphis, TN 38105, USA; 2 Department of Zoology, Jahangirnagar University, Dhaka 1342, Bangladesh; 3 Center of Scientific Excellence for Influenza Viruses, National Research Centre, Giza 12311, Egypt; 4 Department of Epidemiology, Human Genetics and Environmental Sciences, University of Texas Health Sciences Center, Houston, TX 77459, USA; 5 Human Link, Hazmieh, Baabda 1107-2090, Lebanon; 6 National Primate Research Center, University of Washington, Seattle, WA 98195, USA

**Keywords:** avian influenza A virus, domestic duck, migratory bird, reassortment, surveillance

## Abstract

Highly pathogenic avian influenza H5N1 viruses were first isolated in Bangladesh in February 2007. Subsequently, clades 2.2.2, 2.3.4.2 and 2.3.2.1a were identified in Bangladesh, and our previous surveillance data revealed that by the end of 2014, the circulating viruses exclusively comprised clade 2.3.2.1a. We recently determined the status of circulating avian influenza viruses in Bangladesh by conducting surveillance of live poultry markets and waterfowl in wetland areas from February 2015 through February 2016. Until April 2015, clade 2.3.2.1a persisted without any change in genotype. However, in June 2015, we identified a new genotype of H5N1 viruses, clade 2.3.2.1a, which quickly became predominant. These newly emerged H5N1 viruses contained the hemagglutinin, neuraminidase and matrix genes of circulating 2.3.2.1a Bangladeshi H5N1 viruses and five other genes of low pathogenic Eurasian-lineage avian influenza A viruses. Some of these internal genes were closely related to those of low pathogenic viruses isolated from ducks in free-range farms and wild birds in a wetland region of northeastern Bangladesh, where commercially raised domestic ducks have frequent contact with migratory birds. These findings indicate that migratory birds of the Central Asian flyway and domestic ducks in the free-range farms in Tanguar haor-like wetlands played an important role in the emergence of this novel genotype of highly pathogenic H5N1 viruses.

## INTRODUCTION

Highly pathogenic avian influenza (HPAI) H5N1 viruses emerged as a human pathogen in 1997 in Hong Kong.^1^ Since that outbreak, H5N1 viruses spread out of East Asia, across Eurasia, and as far as England and West Africa. H5N1 viruses have caused dramatic economic losses to the poultry industries of many countries. The viruses are also of great concern for public health. Since the reemergence of H5N1 viruses in 2003, 856 laboratory-confirmed cases of human infection, including at least 452 deaths, were reported to the World Health Organization.^2^

The first HPAI H5N1 virus detected, A/Goose/Guangdong/1/1996(H5N1), was identified in 1996 in Guangdong, China and is considered the progenitor H5N1 virus from which present panzootic H5N1 viruses have evolved.^3^ However, contemporary H5N1 viruses contain only the hemagglutinin (HA) gene derived from the progenitor H5N1 virus. The remaining seven viral genes were acquired by genetic reassortment with other avian influenza viruses.^4^ For the past 20 years, the HA genes of H5N1 viruses have evolved extensively, diverging into 40 clades.^5^ Of those 40 clades, only four are currently circulating: 2.2.1.2, 2.3.2.1a, 2.3.2.1c and 2.3.4.4.^6^

In Bangladesh, HPAI H5N1 viruses were first detected in February 2007. Since then, various clades, including 2.2.2, 2.3.4.2, and 2.3.2.1a, of H5N1 have been identified in Bangladesh. Through our previous avian influenza A virus (AIV) surveillance in Bangladesh live poultry markets (LPMs), we found that by the end of 2014, circulating Bangladeshi H5N1 viruses exclusively belonged to clade 2.3.2.1a.^7, 8^

Bangladesh consists of a broad, deltaic plain with many tributaries, subject to frequent flooding by two major rivers, the Brahmaputra and Ganges. The country is located within two major migratory bird flyways, the Central Asian and East Asian-Australian.^9, 10, 11^ The abundance of shallow coastal wetlands and vast inland wetlands (haors), provide a large reservoir for wildlife, especially waterfowl, which migrate from many parts of Europe and Central Asia to overwinter.^12, 13^ Tanguar haor is a large freshwater lake in a wetland area located in the northeast region (Dharmapasha and Tahirpur upazila of Sunamganj district) of Bangladesh, just below the Himalayan Mountains. Commercially raised ducks in the Tanguar haor region are commonly left to scavenge for food during the day, thereby making frequent contact with wild birds. Hence, resident poultry often contribute to the dispersal of the vast gene pool of AIV.

In Bangladesh, low pathogenic avian influenza (LPAI) viruses are highly dominated by the H9N2 subtype. Hereafter the term LPAI refers only to non-H9N2 LPAI viruses. Although H9N2 and several LPAI virus subtypes of Eurasian lineages cocirculate with H5N1 viruses in Bangladesh,^12^ reassortment between HPAI H5N1 and LPAI viruses is somewhat rare. Reassortant H5N1 viruses containing the matrix (M) gene from H9N2 viruses of Chinese lineage^7^ or the polymerase basic 1 (PB1) gene from H9N2 viruses of Bangladeshi lineage^7, 14, 15^ have been isolated from LPMs in Bangladesh, but these viruses did not persist and quickly disappeared.

Here we describe the emergence of H5N1 viruses of a novel genotype. These newly emerged HPAI H5N1 viruses contain the HA (clade 2.3.2.1a), M and neuraminidase (NA) genes of circulating Bangladeshi H5N1 viruses and five genes from Eurasian-lineage LPAI viruses: the PB1, polymerase basic protein 2 (PB2), polymerase acidic protein (PA), nucleoprotein (NP) and nonstructural protein (NS) genes. We found that some of these internal genes were closely related to those of the LPAI viruses that we isolated from the Tanguar haor area in Bangladesh. We performed timely tracking of gene segments from wild birds to domestic ducks and, subsequently, to birds sold in LPMs.

## MATERIALS AND METHODS

### Ethics and compliance

All animal experiments were conducted in an animal biosafety level-3+ facility at St Jude Children’s Research Hospital, in compliance with the policies set forth by the National Institutes of Health and the Animal Welfare Act and with the approval of the St Jude Animal Care and Use Committee.

### Sample collection

In the Tanguar haor area in Bangladesh, oropharyngeal, cloacal and fecal samples were collected from ducks at free-range duck farms, and fresh fecal and water samples were collected from migratory waterfowl. At five LPMs in or near Dhaka, Bangladesh oropharyngeal, cloacal, fecal and water samples were collected from poultry (chickens, ducks, quail and geese) and poultry cages. Samples were collected as described previously,^7, 8^ stored on wet ice blocks (~4 °C) in the field, and were moved to liquid nitrogen storage within one week. After receiving air carrier approval, bimonthly samples were shipped with collection details to the high security biosafety level-3 facilities at St Jude Children’s Research Hospital for analysis.

### Sample screening and virus isolation

We screened all samples (except those collected in December 2015 and February 2016 from the Tanguar haor) by real-time reverse transcription PCR with universal M gene and H5 HA-specific primers, as described previously,^16^ and real-time PCR positive samples are mentioned as FluA-positive and H5-positive samples throughout the manuscript.

All H5-positive samples were inoculated in 10-day-old embryonated chicken eggs for virus isolation. We previously observed that non-H5, FluA-positive samples from domestic chickens and quail yield mostly H9N2 isolates and that other LPAI isolates are rare. Therefore, we propagated ~10% of selected non-H5, FluA-positive samples representing each sampling period, location and host species. However, for ducks and wild birds, most FluA-positive samples were inoculated in eggs.

### Virus subtyping, genome sequencing and phylogenetic analysis

All virus isolates were subtyped by endpoint reverse transcription PCR and sequencing. Viral RNA extraction and nucleotide sequencing were performed as previously described.^7, 17^ Lasergene software (DNASTAR, Madison, WI, USA) was used for nucleotide sequence analysis. We sequenced all eight gene segments (full-length or near full-length) of 45 selected AIVs. These included 14 LPAI viruses isolated from ducks or wild birds in the Tanguar haor, and 20 HPAI H5N1, nine H9N2, one H6N1, and one H4N6 LPAI viruses isolated from Bangladeshi LPMs. In addition, we sequenced the near full-length six H5 HA and five N1 NA genes of six HPAI H5N1 viruses that were co-isolated with other subtype viruses. We also sequenced the HA and NA genes of eight more H9N2 viruses that we isolated from LPMs. The sequences obtained in this study were deposited in the Influenza Research Database and are available under GenBank accession numbers: KY635437–KY635829.

For phylogenetic analyses, sequences other than those found in this study were retrieved from the National Center for Biotechnology Information Influenza Virus Sequence Database^18^ and the EpiFlu database of the Global Initiative on Sharing All Influenza Data.^19^ The sequences were then aligned, and the ends were trimmed to equal lengths using BioEdit sequence alignment editor software (v.7.0.5).^20^ Phylogenetic relationships were inferred by the neighbor-joining method from 500 boot-strap values; the topology was confirmed by the maximum likelihood method;^21^ and evolutionary analyses were conducted with MEGA 7 software.^22^

### Hemagglutination inhibition assays

Hemagglutination inhibition (HI) assays were performed as previously described.^7^ The panel of antisera used in the HI assays included representatives from the currently circulating genetic lineages of clade 2.3.2.1 in Asia, including Bangladesh. We used 0.5% chicken red blood cells for the assay.

### Chicken studies

We used 6- to 8-week-old, specific-pathogen-free white leghorn chickens (Charles River Laboratories, North Franklin, CT, USA) for *in vivo* studies.^23^ Viruses were diluted in sterile PBS in serial logarithmic dilutions. Three chickens per dilution of each virus were inoculated via the natural route (0.2 mL, intranasal; 0.1 mL, intraocular; 0.1 mL, intraoral; and 0.1 mL, intratracheal) to achieve a 0.5 mL total inoculation volume per bird. Oropharyngeal and cloacal swabs were collected on days 2, 4 and 7 postinfection, and birds were monitored at least twice daily for signs of disease, which were recorded. If any neurologic symptoms or signs of morbidity were present or if any bird was unable to eat or drink, it was humanely killed, according to institutional protocol.^23^ Kaplan–Meier plots of post inoculation survival were generated with GraphPad Prism 5.03 (GraphPad Software, La Jolla, CA, USA).

## RESULTS

### Avian influenza A surveillance in Tanguar haor, Bangladesh

**Figure 1:**
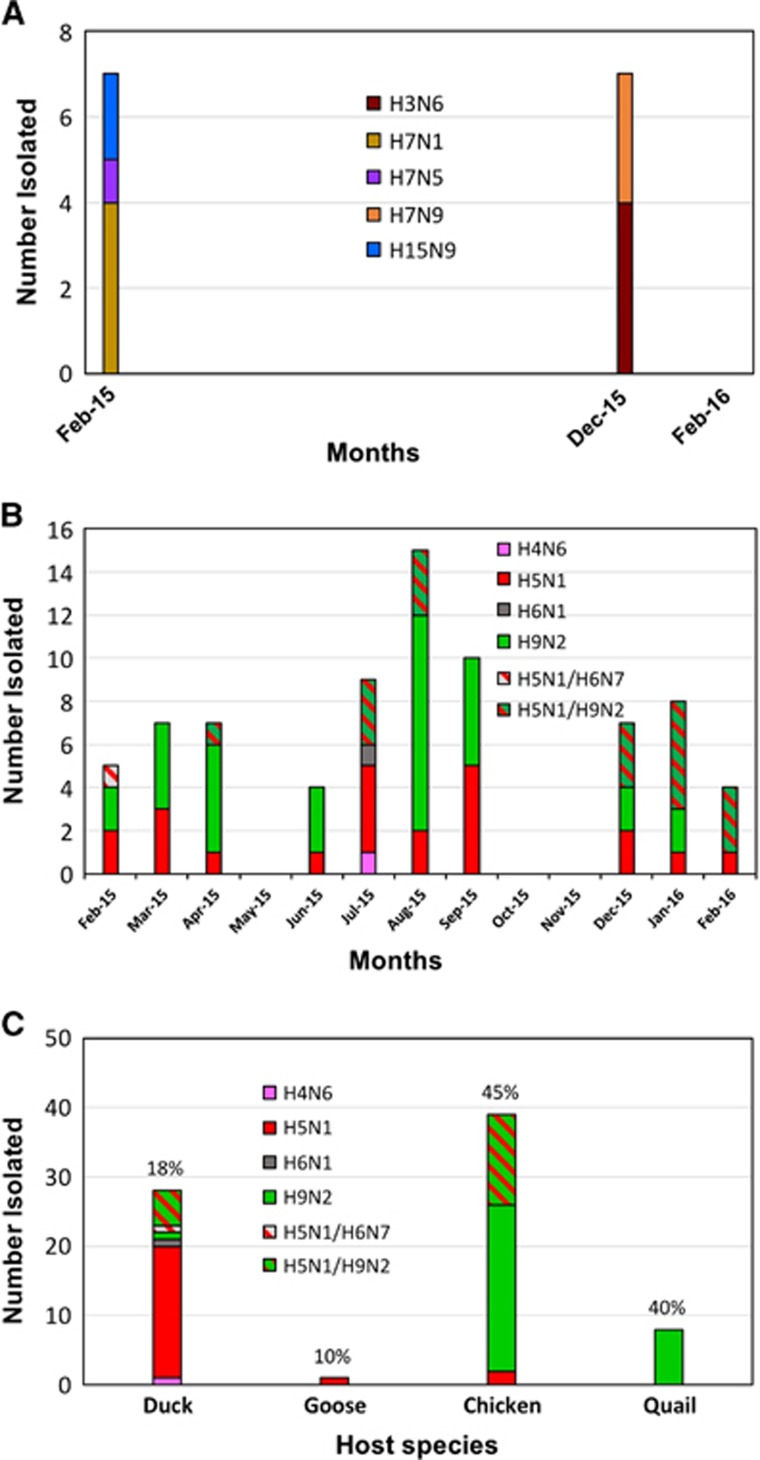
Avian influenza A viruses isolated from (**A**) the Tanguar haor and (**B**) live poultry markets (LPMs) of Bangladesh. (**A**) In February 2015, December 2015 and February 2016, 120, 1000, and 1011 samples, respectively, were collected from ducks (Khaki Campbell) from free-range farms and wild birds in the Tanguar haor area. No viruses were isolated from the samples collected in February 2016. All viruses were isolated from ducks, except one H7N5 virus, which was isolated from a wild black-tailed godwit. (**B**) Monthly virus isolation from LPMs in Bangladesh. From February 2015 through February 2016, 160 samples (95, 95 and 93 samples were collected in February, March and April 2015, respectively) were collected per month from five LPMs in Bangladesh. Samples collected in May, October and November 2015 did not result in any virus isolates. (**C**) Viruses isolated from various species of birds in LPMs. Only 18 samples were collected from geese (April 2015) during the surveillance period, from which one H5N1 virus was isolated. Virus isolation rates (× 100 number of viruses isolated/number of samples inoculated in eggs) are shown on top of each column representing different species.

In February 2015, we collected 55 oropharyngeal/fecal samples from four free-range duck farms and 65 fecal/water samples from migratory waterfowl in the Tanguar haor of Bangladesh. In December 2015, we collected 775 oropharyngeal/cloacal samples from 10 duck farms and 225 fecal samples from wild waterfowl. In February 2016, we collected 511 oropharyngeal/cloacal samples from 22 duck farms and 500 waterfowl fecal samples. We isolated four H3N6, four H7N1, one H7N5, three H7N9 and two H15N9 LPAI viruses from the samples collected in February and December 2015 (Figure 1A). No viruses were isolated from the samples collected in February 2016. All of the viruses were isolated from ducks except for a single H7N5 virus (A/black-tailed godwit/Bangladesh/24734/2015), which was isolated from a fecal sample of a migratory black-tailed godwit (the host species was confirmed by PCR analysis of the fecal sample).

### Avian influenza A surveillance in Bangladesh live poultry markets

We collected 95, 95 and 93 virologic samples (oropharyngeal, cloacal, fecal and water samples) in February, March and April 2015, respectively, from poultry (chickens, ducks, quail and geese) and poultry cages at five LPMs in or near Dhaka, Bangladesh. Thereafter, we collected 160 samples every month until the end of the surveillance period (February 2016). During the study period, we isolated mostly H5N1 and H9N2 along with one H4N6 and one H6N1 subtype AIVs from the LPMs of Bangladesh (Figure 1B). Samples collected in May, October and November 2015 did not result in any virus isolates. However, in May, the prevalence of FluA-positive samples was grossly similar to that observed in other months (average 45%, 42% and 56% FluA-positive samples for ducks, chickens and quail, respectively). For ducks and chickens (but not for quail), the number of FluA-positive samples collected was relatively lower in October (36% and 35% for ducks and chickens, respectively) and November (26% and 29% for ducks and chickens, respectively). The greatest number of H9N2 viruses was isolated in August 2015. However, we did not observe a specific pattern of seasonality for H5N1 or H9N2 viruses (Figure 1B). In Bangladesh, LPAI viruses are highly dominated by H9N2 and throughout the manuscript the term LPAI are used only for non-H9N2 LPAI viruses.

We isolated several AIV subtypes from different species of birds in LPMs (Figure 1C). We found H5N1 viruses predominantly in duck samples. In contrast, we isolated most H9N2 viruses from chicken samples. Quail samples contained only H9N2 viruses. Although we collected samples from ducks, chickens and quail on a regular basis, we collected only 18 samples from geese in April 2015, from which one H5N1 virus was isolated (Figure 1C). In Bangladeshi LPMs, virus isolation rates were considerably higher for chickens (45%) and quail (40%) as compared with ducks (18%) or geese (10%).

### Antigenic analysis in hemagglutination inhibition assays

We used World Health Organization reference antisera and post-infection ferret antisera against the viruses previously isolated from Bangladesh to perform HI assays for all H5N1 and H9N2 viruses that we isolated from Bangladeshi LPMs. We did not observe any overall changes in the antigenicity of either H5N1 (Supplementary Table S1) or H9N2 (Supplementary Table S2) viruses isolated during the surveillance period, compared with previously isolated viruses from Bangladeshi LPMs.^7, 8^ H9N2 viruses isolated from quail are antigenically distinct from H9N2 isolated from chickens, which is also consistent with our previous observations.^24^

### HA and NA phylogeny of LPAI viruses

**Figure 2:**
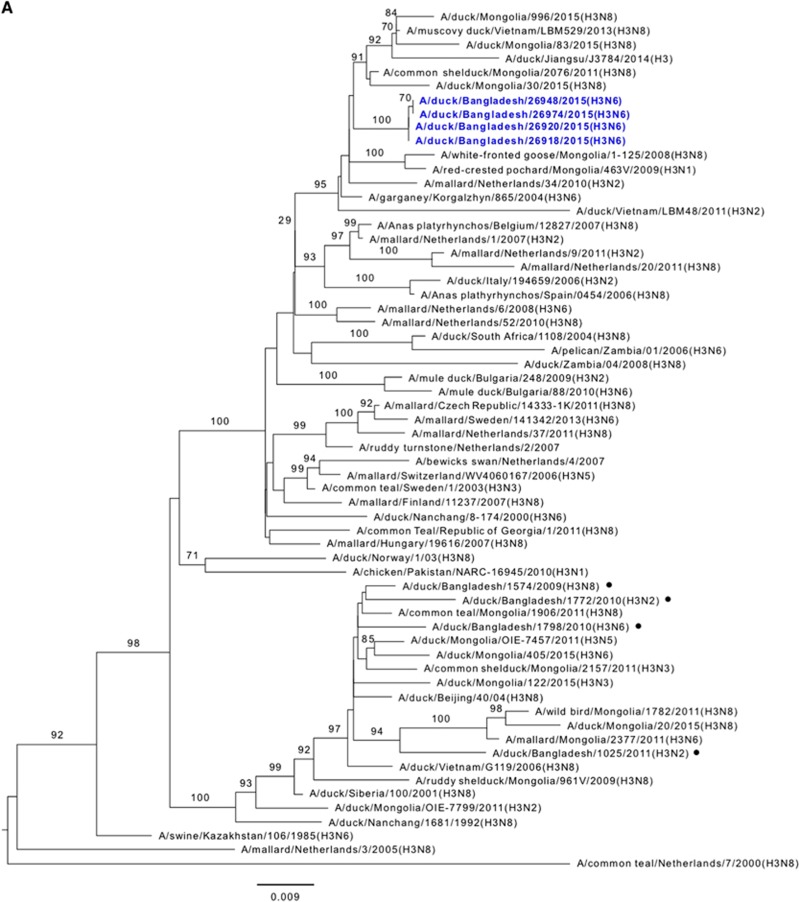

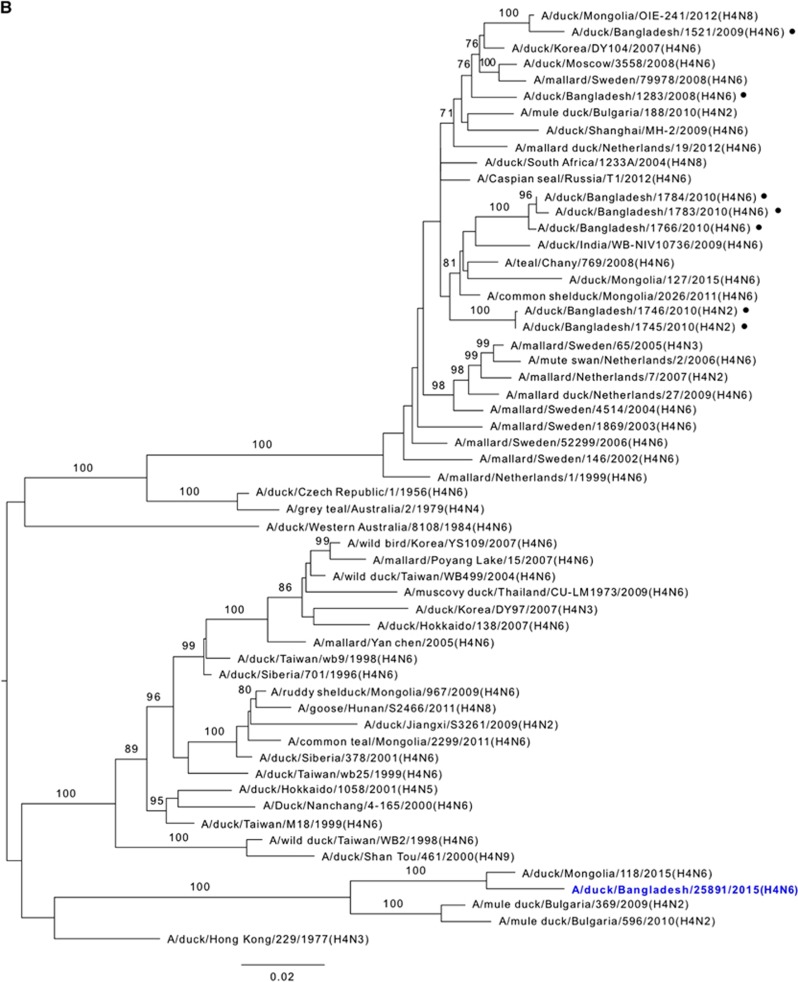

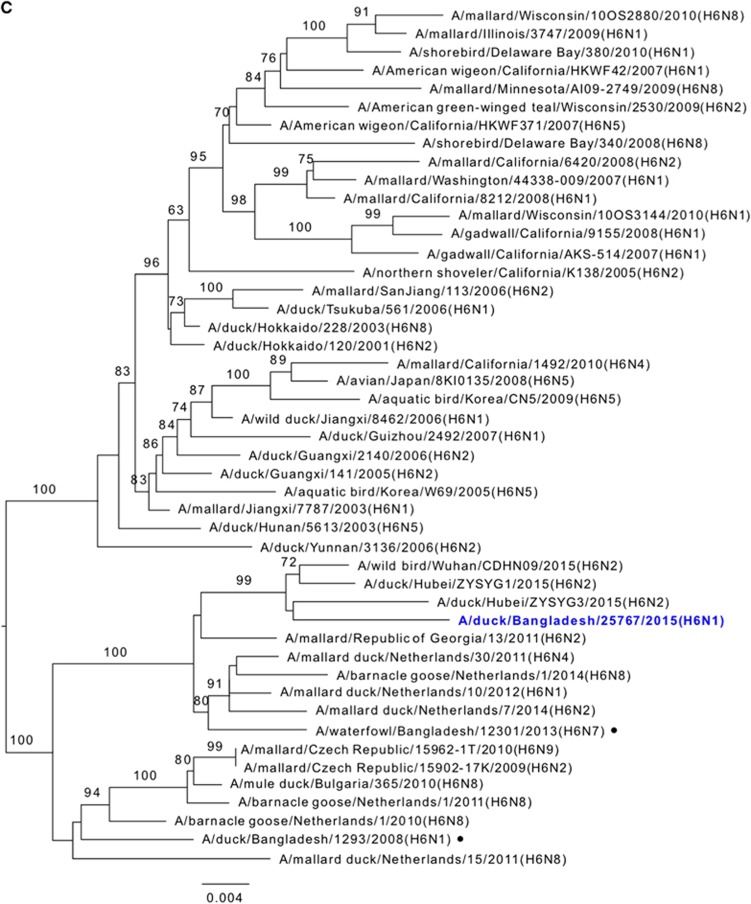

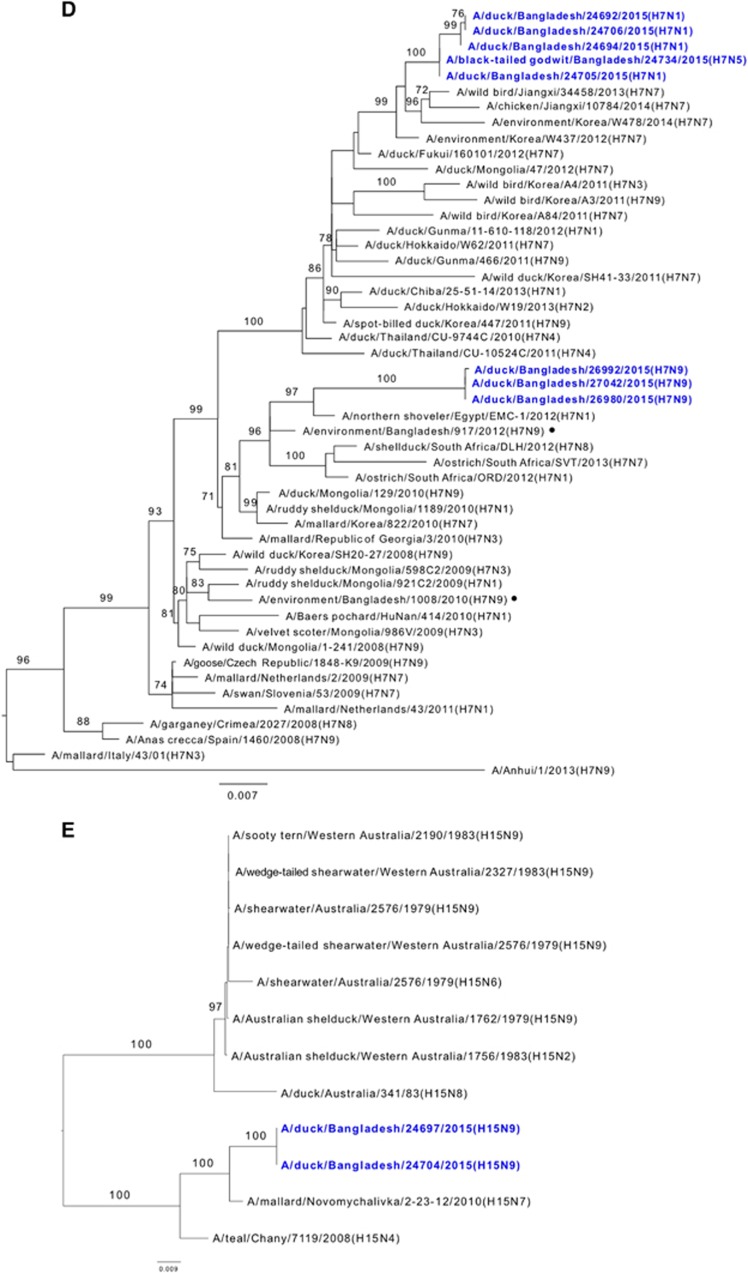
Phylogenetic relationship of the hemagglutinin (HA) genes of (**A**) H3, (**B**) H4, (**C**) H6, (**D**) H7 and (**E**) H15 low pathogenic avian influenza (LPAI) viruses isolated in Bangladesh. Viruses identified during the surveillance period are depicted in blue. •, LPAI viruses previously isolated from Bangladesh. Trees are rooted to midpoint. Bootstrap values ⩾70 are indicated on branches.

Phylogenetic analysis revealed that the HA gene segments of H3, H7 and H15 viruses isolated from ducks and wild birds in the Tanguar haor and H4 and H6 viruses isolated from LPMs were all of Eurasian lineage (Figures 2A–2E). The HA genes of H3 viruses formed a distinct genetic group with those of viruses from the Netherlands, Kazakhstan, Mongolia and Vietnam (Figure 2A). H4 HA is closely related to H4 viruses isolated from Mongolia and Bulgaria (Figure 2B) and H6 HA clustered with H6 viruses isolated from Republic of Georgia and China (Figure 2C). The HA genes of H7 viruses formed two distinct groups in the phylogenetic tree (Figure 2D). In one group, four H7N1 viruses and one H7N5 virus clustered with viruses from South Korea and China (that is, Jiangxi). In the other group, three H7N9 viruses clustered with viruses from South Africa, Egypt and Bangladesh (Figure 2D). Two HA genes of H15N9 viruses clustered with those of viruses isolated from Ukraine (H15N7, Novomychalivka) and Russia (H15N4, Chany) (Figure 2E). We also observed that the NA genes of these viruses were all of Eurasian lineage (Supplementary Figures S1A–S1D). The NA gene segment of H15N9 viruses was distinct from the Australian H15N9 NA and was more closely related to those of H11N9 viruses isolated from Jiangxi, China in 2012 (Supplementary Figure S1D).

### HA and NA phylogeny of H5N1 and H9N2 viruses isolated from Bangladeshi live poultry markets

**Figure 3:**
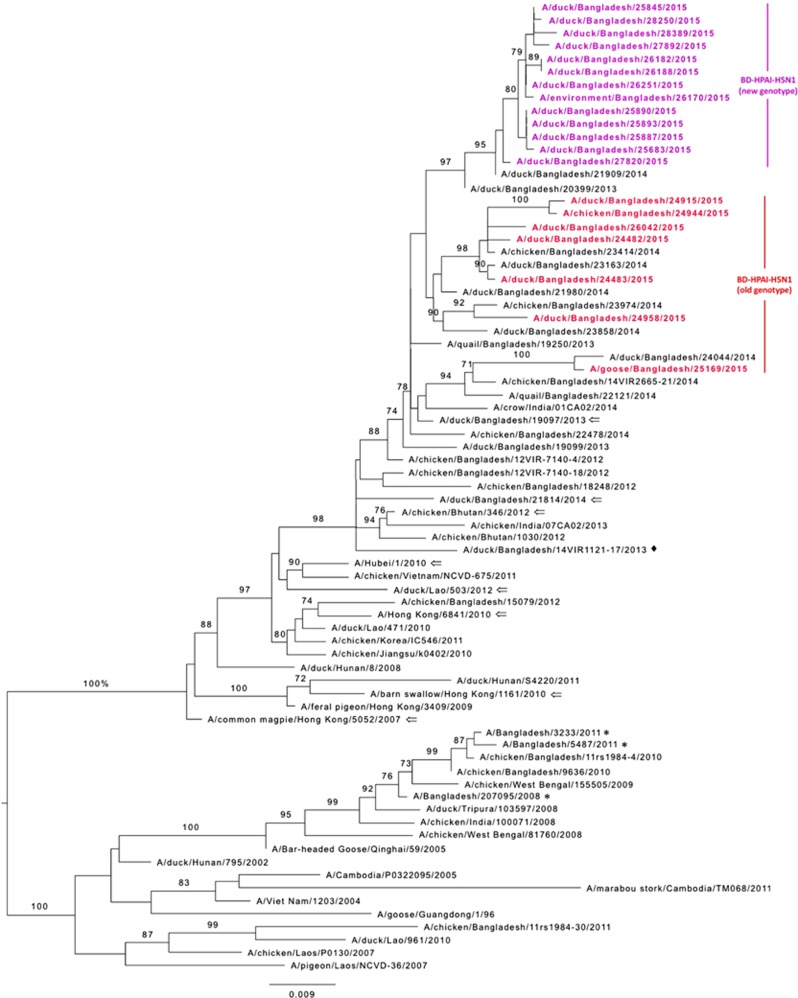
Phylogenetic relationship of the hemagglutinin (HA) genes of the highly pathogenic avian influenza (HPAI) H5N1 viruses isolated from Bangladeshi live poultry markets. The complete coding region of HA1 was used. The viruses identified during the surveillance period are color coded (red and purple for old and new genotype H5N1 viruses, respectively). ♦, viruses isolated in 2013 from Bangladesh with a gene constellation similar to that of new genotype H5N1 viruses described here. *, HPAI H5N1 viruses isolated from humans in Bangladesh. ⇐, reference antigens. Tree is rooted to midpoint. Bootstrap values ⩾70 are indicated on branches.

The H5N1 HA phylogenetic tree revealed that the HA genes of all 20 Bangladeshi H5N1 viruses that we isolated during the surveillance period clustered with clade 2.3.2.1a viruses that were isolated from 2011 through 2014 in Bangladesh, Bhutan and India (Figure 3). Furthermore, the HA genes of 13 recently isolated viruses (purple, Figure 3) formed a monophyletic cluster that shared a common ancestor with those of A/duck/Bangladesh/21909/2014(H5N1). Similar to HA, the NA genes of the same 13 viruses formed a distinct homologous subgroup, sharing a common ancestor with that of A/duck/Bangladesh/21909/2014(H5N1) (purple, Supplementary Figure S2). Phylogenetic analysis of the HA and NA genes of the H9N2 viruses isolated from LPMs in Bangladesh revealed that they were clustered with those of H9N2 viruses that were isolated from Bangladeshi LPMs from 2011 through 2014 (Supplementary Figure S3).

### Phylogeny of internal genes

**Figure 4:**
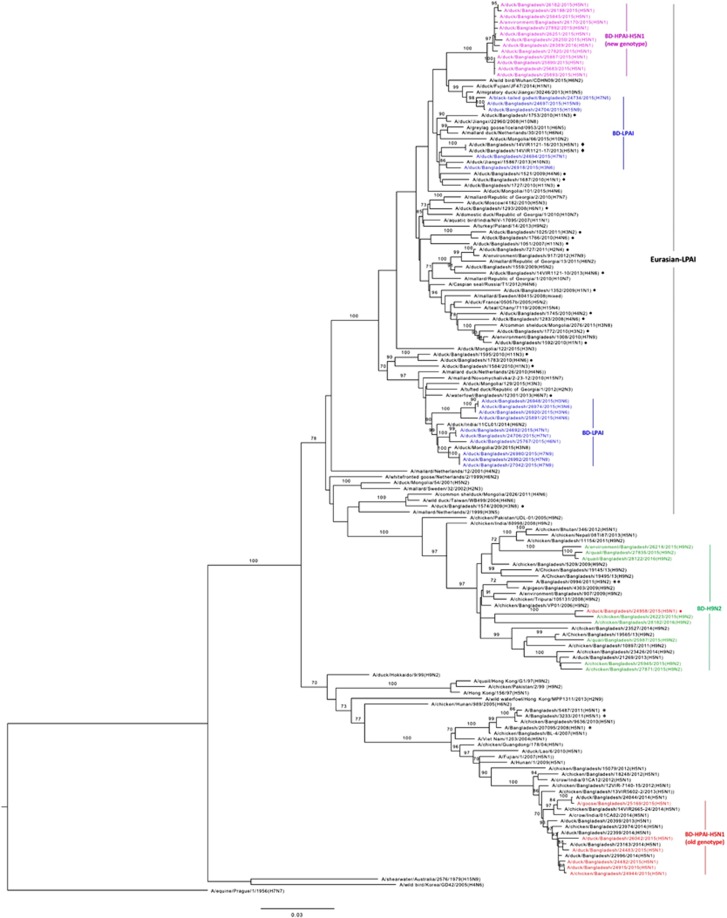
Phylogenetic relationship of the polymerase basic 1 (PB1) genes of the viruses isolated in Bangladesh (BD). The complete coding region, except for first 11 amino acids (2100 nucleotides total), was used. The viruses identified during the surveillance period are color coded (red, old genotype H5N1; purple, new genotype H5N1; green, H9N2; and blue, non-H9N2 LPAI viruses). •, non-H9N2 LPAI viruses previously isolated from Bangladesh. ♦, H5N1 viruses isolated in 2013 from Bangladesh with a gene constellation similar to that of new genotype H5N1 viruses described here. *, HPAI H5N1 viruses isolated from humans in Bangladesh. Tree is rooted to PB1 sequence of A/equine/Prague/1/1956(H7N7). Bootstrap values ⩾70 are indicated on branches.

We determined the phylogeny of all six internal genes of the various subtypes of AIVs that we isolated from Bangladesh. Phylogenetic analysis of PB1 genes segregated the viruses into three major clusters (HPAI H5N1, LPAI H9N2 and non-H9N2 LPAI viruses) (Figure 4). The PB1 genes of the LPAI viruses isolated from the Tanguar haor were all of Eurasian lineage (blue, Figure 4). We isolated four H3N6 viruses from the Tanguar haor, from samples collected in December 2015. The PB1 genes of three of these viruses clustered with those of three H7N9 viruses collected concurrently and with those of two H7N1 viruses collected 10 months earlier (Figure 4). The PB1 gene segments of 10 LPAI viruses isolated from Tanguar hoar and LPMs (three H3N6, one H4N6, one H6N1, two H7N1 and three H7N9) clustered with viruses from Ukraine (Novomychalivka), Mongolia, Republic of Georgia, Bangladesh and India (Figure 4). However, the PB1 genes of the remaining H3N6 virus (A/duck/Bangladesh/26918/2015) clustered separately with that of A/duck/Jiangxi/15867/2013(H10N3). The PB1 genes of two H15N9 viruses and the single H7N5 virus clustered together and were closely related to those of Chinese viruses isolated from Jiangxi (2013/H10N5), Fujian (2014/H1N1) and Wuhan (2015/H6N2) (Figure 4).

The PB1 genes of all H9N2 viruses (green, Figure 4) clustered together with those of contemporary Bangladeshi H9N2 viruses isolated from 2011 through 2014. Surprisingly, the PB1 genes of the HPAI H5N1 viruses isolated from LPMs clustered in two distinct groups. The first group (five isolates collected from February through April 2015 and one isolate collected in August 2015) clustered with the PB1 genes of HPAI H5N1 viruses circulating in Bangladesh and neighboring countries since 2012 (red, Figure 4). However, a second group (13 isolates collected from June 2015 through February 2016) clustered with the PB1 genes of Eurasian-lineage, non-H9N2 LPAI viruses as a monophyletic subgroup (purple, Figure 4), indicating that genetic reassortment of the Bangladeshi HPAI H5N1 viruses occurred with non-H9N2 LPAI viruses of Eurasian lineage. The PB1 gene of only one HPAI H5N1 virus (A/duck/Bangladesh/24958/2015), which was isolated from an LPM in March 2015, clustered with those of H9N2 viruses circulating in Bangladesh. Although rare, reassortment of HPAI H5N1 and H9N2 viruses does occur and has been previously reported by us^7, 8^ and others.^14, 15^

**Figure 5:**
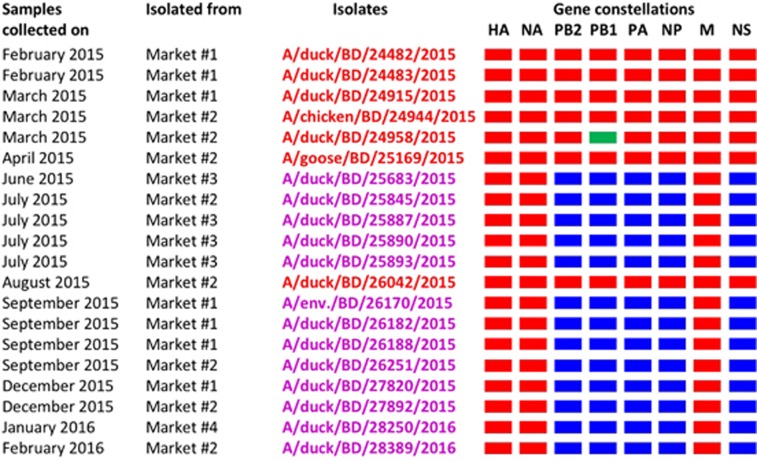
Gene constellations of highly pathogenic avian influenza (HPAI) H5N1 viruses isolated in Bangladesh from February 2015 through February 2016. Gene segments of circulating Bangladeshi HPAI H5N1 (red), Eurasian-lineage (blue), and H9N2-like (green) viruses are depicted for each isolated virus. Note that a newly emerged genotype of H5N1 viruses was first isolated from a single LPM in June 2015. This genotype was then isolated from other markets and became predominant in all Bangladeshi LPMs.

Phylogenetic analysis of other internal genes revealed that the PB2, PA, NP and NS genes of the 13 HPAI H5N1 viruses originated from Eurasian lineages of non-H9N2 LPAI viruses (purple, Supplementary Figure S4A, S4B, S4C, S4E). However, the M genes of these viruses (purple, Supplementary Figure S4D) clustered with those of circulating H5N1 viruses. Similar to the HA and NA genes of the H5N1 viruses, the M genes formed a distinct homologous subgroup that shared a common ancestor with those of A/duck/Bangladesh/20399/2013. Altogether, these results indicate the emergence of a new genotype of HPAI H5N1 viruses. These viruses contained the PB2, PB1, PA, NP and NS genes of Eurasian-lineage LPAI viruses and the HA, NA and M genes of H5N1 viruses that have circulated in Bangladesh since 2012 (Figure 5). We first isolated this new genotype of H5N1 viruses from samples collected from an LPM near Dhaka, Bangladesh in June 2015. In the next few months, we isolated this genotype from samples collected from other LPMs in the Dhaka area. After August 2015, all H5N1 isolates were of the new genotype (Figure 5).

### Pathogenicity in chickens

**Figure 6:**
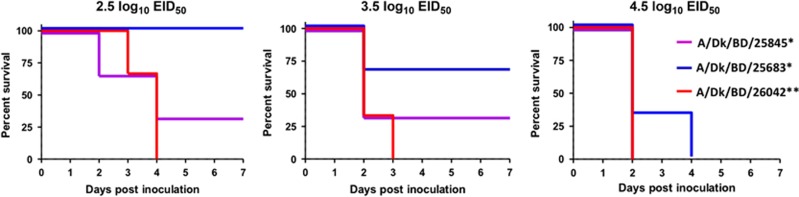
Survival of chickens after inoculation with newly emerged Bangladeshi highly pathogenic avian influenza (HPAI) H5N1 viruses. *, new genotype H5N1 viruses. **, old genotype H5N1 viruses circulating in Bangladesh since 2011. The inoculation doses for the A/Dk/BD/25845 and A/Dk/BD/26042 viruses were 2.5, 3.5 and 4.5 log_10_ EID_50_. The inoculation doses of the A/Dk/BD/25683 virus were 2.67, 3.67 and 4.67 log_10_ EID_50_. Duck, Dk; Bangladesh, BD.

Because of the predominance of the new genotype of HPAI H5N1 viruses in Bangladeshi LPMs, we investigated its pathogenicity in chickens. We determined the 50% chicken lethal dose (CLD_50_) of two new genotypes of HPAI H5N1 viruses (A/duck/Bangladesh/25683/2015 and A/duck/Bangladesh/25845/2015) and one previously circulating HPAI H5N1 (A/duck/Bangladesh/26042/2015, old genotype) virus. The CLD_50_ of A/duck/Bangladesh/25683/2015 and A/duck/Bangladesh/25845/2015 was 2.3 and 3.4 log_10_ 50% egg infectious dose (EID_50_), respectively. In contrast, the CLD_50_ of A/duck/Bangladesh/26042/2015 was 1.7 log_10_ EID_50_, indicating that the newly emerged HPAI H5N1 viruses are of similar or less virulence in chickens than the previously circulating HPAI H5N1 viruses. Accordingly, post-inoculation bird survival was similar for all three HPAI H5N1 viruses (Figure 6).

We observed several signs of disease in infected birds, including ruffled feathers. We frequently observed lethargy between days 2 and 4 post inoculation. Ataxia (evidenced by difficulty walking or perching) was apparent in two birds. We also observed one bird with mild seizures on day 2 post inoculation with A/duck/Bangladesh/26042/2015, which we promptly euthanized.

## DISCUSSION

Domestic ducks are part of an intricate animal production and movement system in Bangladesh. Ducks raised in free-range duck farms in wetland areas have considerable contact with wild migratory birds in production sites, and then with other poultry animals in LPMs. Here, we describe the results of our recent AIV surveillance (February 2015 through February 2016) in poultry traded in LPMs and in wild birds and domestic ducks in the Tanguar haor of Bangladesh. We demonstrated the emergence of H5N1 viruses with a novel genotype. These newly emerged HPAI H5N1 viruses contain the HA, NA, and M genes of HPAI H5N1 viruses previously circulating in Bangladesh and five other genes of Eurasian-lineage LPAI viruses.

The Tanguar haor is a distinct wetland ecosystem that supports 219 species of wild birds (98 migratory and 121 resident). It serves as the major wintering ground of birds migrating in both the Central Asian and Eastern Asian-Australian flyways, with over 16 different migratory duck species, as well as coots and other waterfowl.^13^ The migrating waterfowl first arrive in early November and stay until the end of March. Thus, the free-range duck farm season, from November to May, overlaps with the presence of overwintering migratory waterfowl, resulting in the sharing of the same habitat at the same time.

We isolated 13 LPAI viruses (6 in February 2015 and 7 in the next season, December 2015) from ducks in free-range farms and one from wild birds (February 2015) in the Tanguar haor. Unfortunately, no viruses were isolated in February 2016, though most of the same farms were sampled in December 2015 (same season). Due to lack of systematic studies of virus isolation or serology we cannot explain why we failed to isolate viruses in February 2016. Our findings indicate that Tanguar haor-like ecosystems are a gateway of Eurasian LPAI virus dissemination into and out of Bangladesh. Phylogenetic analyses strongly suggest that continuous reassortment and long distance movement occurs in LPAI viruses.^25^ For example, the HA, NA, PB2, PA, NP and M gene sequences of four H3N6 viruses isolated from free-range duck farms were very similar to each other and were closely related to viruses isolated from Mongolia, Bangladesh, China, Republic of Georgia, Ukraine and the Netherlands. However, the PB1 gene sequence of one of them, A/duck/Bangladesh/26918/2015(H3N6), was distinct and the NS gene sequence was also quite variable from the remaining three viruses.

We observed the emergence of a new genotype of HPAI H5N1 viruses in Bangladesh. These newly emerged HPAI H5N1 viruses contained HA (clade 2.3.2.1a), NA, and M genes that were closely related to those of other HPAI H5N1 viruses circulating in Bangladesh. However, the PB2, PB1, PA, NP and NS genes were more closely related to those of Eurasian-lineage LPAI viruses, although we could not directly attribute these genes to a particular virus. Nevertheless, each gene of the newly emerged viruses formed a distinct monophyletic cluster in their respective phylogenetic trees, indicating that these viruses evolved from a parent virus that reassorted from A/duck/Bangladesh/21909/2014(H5N1)-like Bangladeshi HPAI H5N1 and LPAI viruses of Eurasian linage. It is possible that the newly emerged HPAI H5N1 viruses acquired their five internal genes from a virus that originated after multiple reassortment events among different Eurasian-lineage LPAI viruses.

Inoculation of the newly emerged HPAI H5N1 viruses in chickens revealed that these viruses are of similar or less virulence in chickens compared with the old genotype of H5N1 viruses. A high degree of homogeneity among the HA genes of these viruses and their dominance in LPMs may be beneficial for pandemic preparedness and overall public health concerns. However, we cannot rule out low-frequency co-circulation of the older genotype of HPAI H5N1 viruses. In three months of the surveillance period, we isolated mixtures of H5N1 and H9N2 viruses: three in August 2015, two in January 2016, and one in February 2016. Although the internal genes of these viruses were not sequenced, the H5 HA and N1 NA sequences of two viruses isolated in August 2015 and the two isolated in January 2016 were similar to those of circulating H5N1 (old genotype) viruses (data not shown). Therefore, it is likely that the older genotype H5N1 viruses were circulating, albeit with low frequency, in Bangladeshi LPMs at least until January 2016.

After extensive searching of sequence databases and phylogenetic analyses of all eight gene segments, we found four HPAI H5N1 viruses that belong to a gene constellation similar to the new genotype of H5N1 viruses described here, A/duck/Bangladesh/14VIR1121-12/2013, A/duck/Bangladesh/14VIR1121-16/2013, A/duck/Bangladesh/14VIR1121-17/2013 and A/duck/Bangladesh/14VIR1121-18/2013. A description of these viruses is lacking in the literature. The sequences of all eight viral genes were very similar among each of the four viruses. The PB2 and NS genes of the new genotype of H5N1 viruses shared similarity with those of the four viruses. However, the PB1, PA and NP phylogenetic trees did not reveal a close relation to those of the four viruses. Nonetheless, the NP and PB1 genes of the new genotype of H5N1 viruses (first isolated from an LPM in June 2015) were closely related to A/black-tailed godwit/Bangladesh/24734/2015(H7N5), which was isolated from the Tanguar hoar in February 2015. Therefore, the new genotype of H5N1 viruses described here have recently emerged, likely between February and June 2015, evolving from a parent virus that reassorted from A/duck/Bangladesh/21909/2014(H5N1)-like Bangladeshi HPAI H5N1 and viruses containing Eurasian-linage LPAI genes.

**Figure 7:**
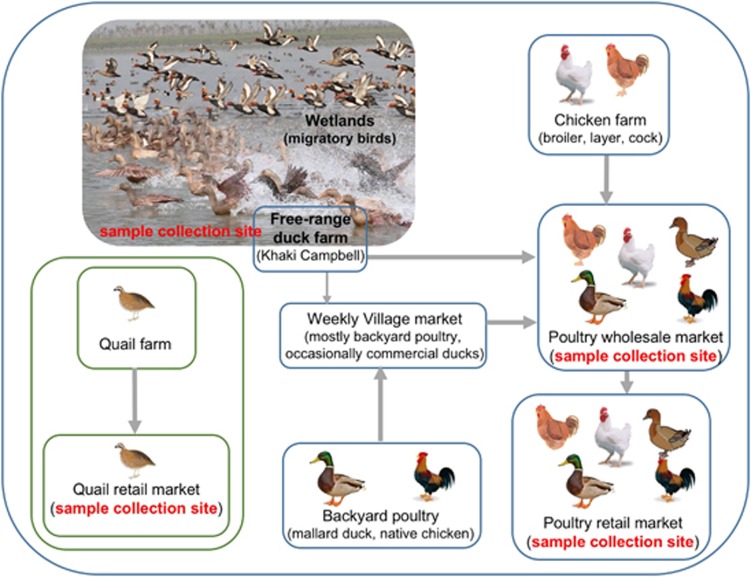
An overview of bird movement in Bangladeshi live poultry markets and sample collection sites (red). Note that quail markets were separate from other poultry markets.

Domestic flocks of Khaki Campbell ducks, ranging from 300 to 1200 birds per flock with an average size around 600 birds, forage for food in sections of the Tanguar haor and are raised in free-range duck farms for egg production. As many as 100 free-range duck farms are present around the edge of the Tanguar haor (field workers' unpublished observation). When it begins to flood in June or July, the natural food supply diminishes, and the ducks from most of the farms are moved to nearby smaller wetlands in either Netrokona or Mymensingh, Bangladesh. Excess ducks are sold through the LPM system. This temporary and/or mobile nature of free-range duck farms at Tanguar hoar may explain the fact that viruses isolated in December 2015 are different from the viruses isolated in the previous season (February 2015). Even when farmers are capable of feeding ducks during the flood season, they do not keep these ducks longer than three years and sell approximately one-third of their flock annually to the LPM system (Figure 7). Therefore, these ducks are most likely the carriers of LPAI viruses, acquiring the viruses from wild migratory birds in the wetlands and transmitting the viruses to LPMs. This hypothesis is supported by the close association among the PB1 and NP genes of the newly emerged HPAI H5N1 and LPAI viruses isolated from free-range duck farms and wild birds in the Tanguar haor. However, we cannot rule out the possibility of virus movement from LPMs to free-range farms in Tanguar haor-like ecosystems, which may occur via contaminated poultry cages or fomites.

Although HPAI H5N1 and LPAI H9N2 viruses cocirculate and coinfect birds in the LPMs in Bangladesh, they rarely produce reassortants. The reassortants that have been detected failed to become established and disappeared. In contrast, the LPAI viruses isolated from migratory waterfowl and domestic duck farms in the Tanguar haor do reassort with circulating HPAI H5N1 viruses. This has generated a new genotype of HPAI H5N1 viruses that are now dominant and represent the current threat to domestic poultry and humans in this region. To date, there have been no reports of these newly emerged H5N1 viruses in humans. Continued surveillance of the LPMs and wetland areas in Bangladesh is necessary for pandemic preparedness and to mitigate overall public health concerns.
