# Supplementary material for: Role of domestic ducks in the emergence of a new genotype of highly pathogenic H5N1 avian influenza A viruses in Bangladesh
Source: Emerg Microbes Infect. 2017 Aug 9;6(8):e72–. doi: 10.1038/emi.2017.60 (PMC5583668; doi:10.1038/emi.2017.60)
Supplement: Supplementary Table S1 [file emi201760x1.docx]

**Supplementary Table S1 Antigenic analysis of H5N1 influenza A viruses from Bangladesh by hemagglutination inhibition assay**

|  |  |  |  |  |  |  |  |  |  |
| --- | --- | --- | --- | --- | --- | --- | --- | --- | --- |
|  |  | **αH5N1 (postinfection ferret antisera)** | | | | | | | |
|  |  |  |  |  |  |  |  |  |  |
|  |  |  |  |  |  |  |  |  |  |
| **H5N1 antigen** | **Clade** | **αCM/HK/5052** | **αHK/6841** | **αBS/HK/1161** | **αHubei/1** | **αDk/Bd/19097** | **αCk/Bd/21814** | **αCk/Bh/346** | **αDk/Lao/503** |
|  |  |  |  |  |  |  |  |  |  |
|  |  |  |  |  |  |  |  |  |  |
| **Reference antigen** |  |  |  |  |  |  |  |  |  |
| rg-A/common magpie/Hong Kong/5052/2007 | 2.3.2.1 | **320** | 320 | >1280 | 20 | 160 | 40 | 80 | 80 |
| A/Hong Kong/6841/2010 | 2.3.2.1c | 80 | **320** | 640 | 20 | 320 | 40 | 160 | 160 |
| rg-A/barn swallow/Hong Kong/1161/2010 | 2.3.2.1b | 20 | 320 | **640** | 10 | 160 | 20 | 80 | 80 |
| rg-A/Hubei/1/2010 | 2.3.2.1a | 320 | 640 | 640 | **320** | 640 | 40 | 160 | 160 |
| A/duck/Bangladesh/19097/2013 | 2.3.2.1a | 80 | 160 | 320 | 10 | **320** | 40 | 160 | 160 |
| A/chicken/Bangladesh/21814/2014 | 2.3.2.1a | 10 | 320 | 80 | 10 | 640 | **320** | 640 | 320 |
| A/chicken/Bhutan/346/2012 | 2.3.2.1a | <10 | 160 | 40 | <10 | 320 | 160 | **320** | 160 |
| A/duck/Lao/503/2012 | 2.3.2.1a | 80 | 160 | 320 | 10 | 320 | 40 | 160 | **160** |
| **Test antigen** |  |  |  |  |  |  |  |  |  |
| A/duck/Bangladesh/24482/2015 | 2.3.2.1a | <10 | 160 | 40 | <10 | 320 | 80 | 320 | 160 |
| A/duck/Bangladesh/24483/2015 | 2.3.2.1a | <10 | 10 | <10 | <10 | 40 | 10 | 40 | 20 |
| A/duck/Bangladesh/24915/2015 | 2.3.2.1a | <10 | 80 | 20 | <10 | 160 | 40 | 160 | 80 |
| A/chicken/Bangladesh/24944/2015 | 2.3.2.1a | <10 | 80 | 20 | <10 | 160 | 40 | 160 | 80 |
| A/duck/Bangladesh/24958/2015 | 2.3.2.1a | 10 | 40 | 40 | <10 | 80 | <10 | 40 | 40 |
| A/goose/Bangladesh/25169/2015 | 2.3.2.1a | 40 | 160 | 160 | 10 | 160 | 20 | 80 | 80 |
| A/duck/Bangladesh/25683/2015 | 2.3.2.1a | 80 | 160 | 320 | 10 | 160 | 20 | 160 | 160 |
| A/duck/Bangladesh/25845/2015 | 2.3.2.1a | 80 | 320 | 320 | 10 | 320 | 40 | 160 | 160 |
| A/duck/Bangladesh/25887/2015 | 2.3.2.1a | 40 | 80 | 160 | 10 | 160 | 20 | 160 | 160 |
| A/duck/Bangladesh/25890/2015 | 2.3.2.1a | 40 | 80 | 160 | <10 | 160 | 20 | 80 | 80 |
| A/duck/Bangladesh/25893/2015 | 2.3.2.1a | 40 | 80 | 160 | <10 | 160 | 20 | 80 | 80 |
| A/duck/Bangladesh/26042/2015 | 2.3.2.1a | <10 | 20 | 10 | <10 | 160 | 20 | 80 | 80 |
| A/environment/Bangladesh/26170/2015 | 2.3.2.1a | 80 | 80 | 320 | 10 | 20 | 10 | 20 | 40 |
| A/duck/Bangladesh/26182/2015 | 2.3.2.1a | 40 | 80 | 160 | <10 | 80 | 10 | 80 | 80 |
| A/duck/Bangladesh/26188/2015 | 2.3.2.1a | 20 | 80 | 160 | <10 | 80 | 20 | 80 | 40 |
| A/duck/Bangladesh/26251/2015 | 2.3.2.1a | 40 | 160 | 320 | <10 | 160 | 20 | 80 | 160 |
| A/duck/Bangladesh/26253/2015 | 2.3.2.1a | 80 | 160 | 320 | <10 | 160 | 20 | 80 | 80 |
| A/duck/Bangladesh/27820/2015 | 2.3.2.1a | 40 | 80 | 160 | <10 | 160 | 20 | ND | ND |
| A/duck/Bangladesh/27892/2015 | 2.3.2.1a | 10 | 160 | 80 | 10 | 320 | 20 | ND | ND |
| A/duck/Bangladesh/28250/2015 | 2.3.2.1a | 40 | 160 | 160 | <10 | 320 | 20 | ND | ND |
| A/duck/Bangladesh/28389/2015 | 2.3.2.1a | 20 | 80 | 80 | <10 | 160 | 20 | ND | ND |

Abbreviations: Bd, Bangladesh; Bt, Bhutan; BS, barn swallow; Ch, chicken; CM, common magpie; Dk, duck; HK, Hong Kong; ND, not determined; rg, reverse genetic.

Titers are expressed as the reciprocal of the highest dilution of the last dilution that completely inhibited hemagglutination of 0.5% chicken erythrocytes. Boldface/underline indicates homologous serum.
