# Supplementary material for: Role of domestic ducks in the emergence of a new genotype of highly pathogenic H5N1 avian influenza A viruses in Bangladesh
Source: Emerg Microbes Infect. 2017 Aug 9;6(8):e72–. doi: 10.1038/emi.2017.60 (PMC5583668; doi:10.1038/emi.2017.60)
Supplement: Supplementary Table S2 [file emi201760x2.docx]

**Supplementary Table S2 Antigenic analysis of H9N2 influenza A viruses from Bangladesh by hemagglutination inhibition assay**

|  |  |  |  |  |  |  |  |  |  |  |
| --- | --- | --- | --- | --- | --- | --- | --- | --- | --- | --- |
|  |  |  | **αH9N2 (post-infection ferret antisera)** | | | | | | | |
|  |  |  |  |  |  |  |  |  |  |  |
|  |  |  |  |  |  |  |  |  |  |  |
| **H5N1 antigen** | **Lineage** |  | **αHK/1073** | **αBd/0994** | **αQu/Bd/19462** | **αCk/Eg/D74388** | **αCk/Bd/21940** | **αCk/HK/G9** | **αHK/33982** | **αHK/308** |
|  |  |  |  |  |  |  |  |  |  |  |
|  |  |  |  |  |  |  |  |  |  |  |
| **Reference antigen** |  |  |  |  |  |  |  |  |  |  |
| A/Hong Kong/1073/97 | G1 |  | **640** | 10 | 10 | 10 | 10 | 20 | 160 | <10 |
| A/Bangladesh/0994/2011 | G1 |  | 80 | **2560** | 320 | 640 | 640 | 160 | 40 | 20 |
| A/quail/Bangladesh/19462/2013 | G1 |  | 160 | 320 | **320** | 320 | 80 | 160 | 20 | 20 |
| A/chicken/Egypt/D7438B/2013 | G1 |  | 160 | 320 | 160 | **2560** | 320 | 320 | 40 | 40 |
| A/chicken/Bangladesh/21940/2014 | G1 |  | 80 | 640 | 320 | 640 | **640** | 160 | 40 | 20 |
| A/chicken/Hong Kong/G9/97 | Y280 |  | 80 | 320 | 80 | 320 | 160 | **640** | 20 | 80 |
| A/Hong Kong/33982/2009 | Y280 |  | 160 | <10 | <10 | <10 | <10 | <10 | **1280** | <10 |
| A/Hong Kong/308/2014 | Y280 |  | 80 | 40 | 80 | 40 | 40 | 80 | 40 | **5120** |
| **Test antigen** |  |  |  |  |  |  |  |  |  |  |
| A/chicken/Bangladesh/25945/2015 | G1 |  | 160 | 1280 | 640 | 1280 | 1280 | 320 | 80 | 40 |
| A/chicken/Bangladesh/26024/2015 | G1 |  | 80 | 2560 | 640 | 1280 | 640 | 160 | 40 | 20 |
| A/chicken/Bangladesh/26102/2015 | G1 |  | 160 | 1280 | 80 | 1280 | 320 | 320 | 40 | 20 |
| A/chicken/Bangladesh/26115/2015 | G1 |  | 160 | 1280 | 320 | 2560 | 640 | 320 | 80 | 20 |
| A/chicken/Bangladesh26120/2015 | G1 |  | 160 | 1280 | 320 | 1280 | 320 | 320 | 80 | 40 |
| A/chicken/Bangladesh/26223/2015 | G1 |  | 160 | 5120 | 160 | 1280 | 640 | 320 | 80 | 20 |
| A/chicken/Bangladesh/26231/2015 | G1 |  | 80 | 1280 | 640 | 1280 | 640 | 320 | 40 | 20 |
| A/chicken/Bangladesh/26238/2015 | G1 |  | 160 | 2560 | 320 | 1280 | 640 | 320 | 40 | 40 |
| A/chicken/Bangladesh/27871/2015 | G1 |  | 80 | 5120 | 640 | 1280 | 640 | 320 | 80 | 40 |
| A/chicken/Bangladesh/28182/2015 | G1 |  | 80 | 640 | 320 | 640 | 160 | 160 | 20 | 20 |
| A/environment/Bangladesh/25969/2015 | G1 |  | 80 | 5120 | 80 | 1280 | 640 | 320 | 20 | 20 |
| A/environment/Bangladesh/26032/2015 | G1 |  | 80 | 2560 | 320 | 640 | 640 | 160 | 40 | 20 |
| A/environment/Bangladesh/26038/2015 | G1 |  | 160 | 2560 | 1280 | 2560 | 1280 | 640 | 80 | 20 |
| A/environment/Bangladesh/26176/2015 | G1 |  | 160 | 5120 | 1280 | 1280 | 640 | 320 | 80 | 20 |
| A/environment/Bangladesh/26243/2015 | G1 |  | 160 | 2560 | 1280 | 2560 | 640 | 320 | 160 | 80 |
| A/environment/Bangladesh/26244/2015 | G1 |  | 320 | 2560 | 1280 | 1280 | 640 | 640 | 80 | 40 |
| A/quail/Bangladesh/25987/2015 | G1 |  | 20 | 40 | 80 | 40 | 20 | 20 | <10 | 10 |
| A/quail/Bangladesh/25992/2015 | G1 |  | 40 | 40 | 80 | 80 | 40 | 20 | <10 | 10 |
| A/quail/Bangladesh/25997/2015 | G1 |  | 20 | 40 | 80 | 40 | 20 | 20 | <10 | <10 |
| A/quail/Bangladesh/27835/2015 | G1 |  | 40 | 80 | 160 | 40 | 20 | 20 | 40 | 10 |
| A/quail/Bangladesh/28122/2015 | G1 |  | 80 | 80 | 160 | 80 | 40 | 40 | 40 | 20 |
| A/environment/Bangladesh/26218/2015 | G1 |  | 80 | 40 | 160 | 80 | <10 | 40 | 40 | 10 |

Abbreviations: Bd, Bangladesh; Ck, chicken; Eg, Egypt; HK, Hong Kong; Qu, quail.

Titers are expressed as the reciprocal of the highest dilution of the last dilution that completely inhibited hemagglutination of 0.5% chicken erythrocytes. Boldface/ underline indicates homologous serum.
